# Editorial: Fruit ripening: From present knowledge to future development, Volume II

**DOI:** 10.3389/fpls.2022.1078841

**Published:** 2022-12-01

**Authors:** José M. Palma, Francisco J. Corpas, Luciano Freschi

**Affiliations:** ^1^ Group of Antioxidants, Free Radicals and Nitric Oxide in Biotechnology, Food and Agriculture, Estación Experimental del Zaidín, Consejo Superior de Investigaciones Científicas (CSIC), Granada, Spain; ^2^ Departamento de Botânica, Instituto de Biociências, Universidade de São Paulo, São Paulo, Brazil

**Keywords:** cutting-edge technologies, fruit, omics, quality, ripening

Fruits are a common component of the human nutrition worldwide. Thus, it is well known that fruits not only provide sugars, polyunsaturated fatty acids (PUFA), fiber and other macro-elements to our diet, but also essential metabolites such as vitamins and antioxidants. These include vitamins A (synthesized from β-carotene), C (ascorbic acid) and E (α-tocopherol), carotenoids (carotenes plus xanthophylls) and polyphenols, among others. Additionally, fruits are also a source of diverse secondary metabolites with bioactive functions and therapeutic potential in human.

Thus, capsaicin, an alkaloid exclusive of the pungency of hot peppers (*Capsicum annuum*), exhibits analgesic properties, but it also shows some functions in the cardiovascular, nervous and immune systems against infectious diseases, in inflammation, obesity and cancer (Sancho et al., 2002; Clark and Lee, 2016; Guevara et al., 2021; Merritt et al., 2022). Vincristine and its analog, vinblastine, are alkaloids obtained from Cape periwinkle, also called graveyard plant and bright eyes (*Catharanthus roseus*), that are employed in chemotherapy as an additional medication in treating pathologies such as the Hodgkin’s lymphoma and other cancers including the non-small cell lung, bladder, brain, melanoma, and testicular cancer (Martino et al., 2018; Schiller et al., 2018; Tsubaki et al., 2018; Zhou et al., 2019). Flavonoids (anthocyanins and quercetin, among others) are antioxidant metabolites of plant origin present in fruits that also have therapeutic relevance. Clinical studies have proven that anthocyanins show anti-inflammatory, antiobesity, cardio- and neuro-protective properties, preventive effects in conditions such as diabetes and cancer, and therapeutic uses in Alzheimer’s disease (Salehi et al., 2020; Suresh et al., 2022). Likewise, in *in vitro* assays with animal models, quercetin displays antiviral, anti-inflammatory, and anti-tumoral activities, and reduces capillary permeability and platelet aggregation due to its capacity to inhibit cyclooxygenases and lipoxygenases (Costa et al., 2016; Reyes-Farias and Carrasco-Pozo, 2019; Chen et al., 2020; Li et al., 2022). The list of plant-derived metabolites with pharmacological and therapeutic potential continues to grow.

Therefore, fruits, besides being viewed as attractive food that decorates our markets and dishes and adds appealing aromas, delicious taste, and essential macronutrients to our diet, should also be envisaged as vectors of nutraceutical products that may contribute to a better health status. Thus, as depicted in **Figure 1**, ongoing efforts aiming to improve our knowledge of fruit physiology will contribute to providing added value to fruit quality. Such efforts will benefit from synergistic interactions among academic researchers, industry partners, growers/farmers and consumers, driving the iterative process of continuous improvement of the quality and productivity of our crops.

**Figure 1:**
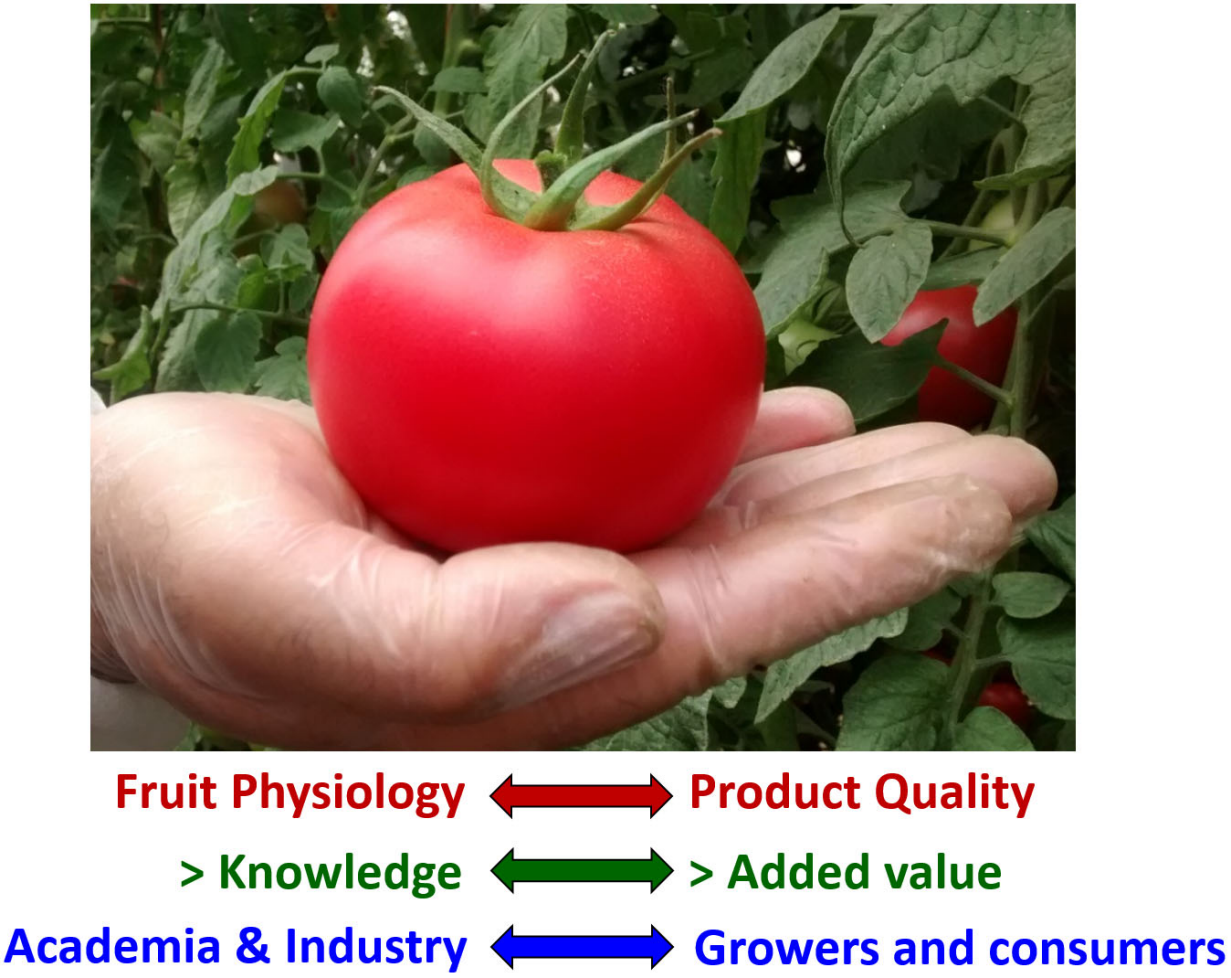
Synergistic interactions between the academia, industry, growers, and consumers will guide future efforts towards improving fruit productivity, flavor, and nutritional quality.

With this perspective in mind, the Research Topic on Fruit Ripening: From Present Knowledge to Future Development was organized. This second volume provides new insights at the intersection of the human search for tastier and more nutritious food and cutting-edge technologies to advance our knowledge on fruit ripening and improve sensorial and nutritional quality to promote human health.

Thus, for example, the use of modern, commercial-scale Protected Cropping Systems (PCS) on the sweet cherry (*Prunus avium*) orchard microclimate and their effects on tree water uptake and fruit quality have been investigated, highlighting the agronomical implications of partially controlled environmental conditions on this fruit crop (Stone et al.). Transcriptomic approaches have been applied to study diverse aspects of strawberry, melon and citrus fruit physiology. In strawberry (*Fragaria × ananassa* Duch.), the analysis of the temporal changes in alternative transcription start or termination sites (aTSS or aTTS, respectively) as well as alternative splicing (AS) events, which produce diverse transcript isoforms, has provided a new comprehensive overview of the dynamic transcriptomic landscape during fruit development and maturation (Chen et al.). On the other hand, integrated transcriptomic and metabolomic analyses have widened the information on the key gene networks controlling the soluble sugar and organic acid metabolisms during oriental melon (*Cucumis melo* var. *acidulus*) fruit development (Cheng et al.). Transcriptomic and other molecular, cellular and analytical techniques have also been used as tools to elucidate the genetic and molecular regulation of citric acid synthesis and degradation in fruits of the citrus Ponkan (*Citrus reticulata* Blanco cv. Ponkan) (Liu et al.). And the combination of transcriptomics and physiological analysis in Satsuma mandarin (*Citrus unshiu* Marc.) has also taught us how low temperatures could be involved, in an ethylene-independent manner, in controlling ripening-related fruit peel degreening (Mitalo et al.).

Development and ripening are, perhaps, the most important events influencing fruit appearance and quality, and these physiological processes have been tackled from different perspectives in this Research Topic. In tomato (*Solanum lycopersicum*), new insights into the orchestrated environmentally-modulated epigenetic processes affecting agronomical traits have been obtained through the integrative analysis of the methylome, transcriptome and sRNAome in fruits of phytochrome-deficient mutants (Bianchetti et al.). Overall, fruit ripening involves a series of phenotypic, physiological and biochemical changes. These include, among others, alterations in the fruit color as a consequence of the chlorophyll degradation and the synthesis of new carotenoids and anthocyanins; cell wall remodeling, which affects fruit softening and texture; and changes in the profile of sugars, organic acids and secondary metabolites, such as volatiles, which influence fruit flavor and aroma. Accumulating evidence indicates that the coordinated developmental and metabolic changes during fruit ripening depend on a complex regulatory network consisting of transcription factors, co-regulators, hormonal signals, and epigenetic modifications. In this issue, an overview of the current knowledge on the role of transcription factors and candidate regulators in modulating strawberry fruit ripening has been provided (Sánchez-Gómez et al.). Additionally, the role in different plant species of the AP2/ERF (APETALA2/ETHYLENE RESPONSIVE FACTOR) transcription factors in the formation of key fruit-ripening attributes, their regulatory action by interacting with other proteins, their role in orchestrating the phytohormone-signaling networks, and the epigenetic modifications associated with their gene expression are reviewed and discussed in this volume (Zhai et al.).

We hope this Research Topic provides a useful compilation of recent studies and a prospective view in the area of fruit ripening and encourages more researchers to work in this exciting field of plant biology.

## Author contributions

The authors conceived, contributed to write, corrected and approved the manuscript.

## Funding

JMP and FJC were supported by European Regional Development Fund-cofinanced grants from the Ministry of Science and Innovation (PID2019-103924GB-I00) and Junta de Andalucía (P18-FR-1359), Spain. LF was supported by São Paulo Research Foundation (2018/16389-8, 2016/04924-0, 2017/17935-3, 2016/01128-9), Conselho Nacional de Desenvolvimento Científico e Tecnológico (422287/2018-0, 305012/2018-5, 303332/2019-0,300986/2018-1), and Coordenação de Aperfeiçoamento de Pessoal de Nível Superior, Brazil. 

## Conflict of interest

The authors declare that the research was conducted in the absence of any commercial or financial relationships that could be construed as a potential conflict of interest.

## Publisher’s note

All claims expressed in this article are solely those of the authors and do not necessarily represent those of their affiliated organizations, or those of the publisher, the editors and the reviewers. Any product that may be evaluated in this article, or claim that may be made by its manufacturer, is not guaranteed or endorsed by the publisher.
